# Lung transplantation for chronic thromboembolic pulmonary hypertension

**DOI:** 10.1016/j.jhlto.2025.100461

**Published:** 2025-12-03

**Authors:** Laurent Godinas, Michaela Orlitová, Emanuele Muscogiuri, Laurens Ceulemans, Elie Fadel, Marion Delcroix, Tom Verbelen

**Affiliations:** a Department of Respiratory Diseases, University Hospitals Leuven, Leuven, Belgium; b Laboratory of Respiratory Diseases and Thoracic Surgery (BREATHE), Department of Chronic Diseases and Metabolism, KU Leuven, Leuven, Belgium; c Department of Thoracic Surgery, University Hospitals Leuven, Leuven, Belgium; d Department of Cardiovascular Sciences, KULeuven, Leuven, Belgium; e Department of Radiology, University Hospitals Leuven, Leuven, Belgium; f Thoracic Surgery and Transplantation Department, Centre Chirurgical Marie Lannelongue, Le Plessis-Robinson, France; g Department of Cardiac Surgery, University Hospitals Leuven, Leuven, Belgium

**Keywords:** Chronic thromboembolic pulmonary hypertension, Pulmonary hypertension, Lung transplantation, Pulmonary endarterectomy, Balloon pulmonary angioplasty

## Abstract

Therapeutic advances in chronic thromboembolic pulmonary hypertension (CTEPH) over the last 10 years have improved patient prognosis. As a result, the role of lung transplantation (LTx) for this indication has been greatly reduced. In this review article, we describe the evolution of treatments for CTEPH, the place that remains for lung transplantation, and the particularities of lung transplantation for this indication.

## Background

The last 10 years have seen major advances in the treatment of chronic thromboembolic pulmonary hypertension (CTEPH). Alongside pulmonary endarterectomy (PEA) surgery, new treatments have been developed. Firstly, medical treatments have been shown to be effective in clinical trials. Riociguat demonstrated its efficacy in a randomized, controlled, double-blind study.^1^ Treprostinil and macitentan have also shown their efficacy in CTEPH.^2^, ^3^ Moreover, balloon pulmonary angioplasty (BPA) has gradually gained in importance, showing its efficacy on different outcomes as hemodynamics, functional capacity, symptoms, quality of life, and right ventricular (RV) function.^4^ Data from various registries also show an improvement in survival, with nowadays comparable survival in patients treated with BPA or PEA.^5^ All these treatments have benefited inoperable patients, either for technical reasons (distal lesions) or because of co-morbidities precluding PEA, but also patients for whom surgery failed or had an inadequate result after PEA. Moreover, with a better understanding of the disease, physiology, enhanced perioperative management, and improved surgical techniques, PEA itself has now become a low-risk procedure with acceptable low mortality and morbidity, even in high-risk patients.^6^, ^7^, ^8^ In contrast to the past, a challenging distal endarterectomy is now mastered by more and more experienced PEA surgeons. The use of currently available fine instruments allows to reach disease located in the lumen of subsegmental vessels, or alternatively, the surgeon can start the dissection plane more proximal below the normal fragile intima and meticulously continue this until diseased intima is reached that then can be removed.

The percentage of patients still suffering from severe and life-threatening disease has therefore fallen sharply over the last 10 years. The role of lung transplantation (LTx), which used to be the only possible treatment for certain well-selected CTEPH patients, has significantly reduced. In this review, we will address the remaining place for LTx in CTEPH, its modalities, and outcomes.

## CTEPH: Definition, epidemiology, and physiopathology

CTEPH is a disease defined by the presence of symptoms associated with precapillary pulmonary hypertension (PH), defined as mean pulmonary artery pressure > 20 mmHg with pulmonary vascular resistance (PVR) > 2 WU, and persistent perfusion defects after at least 3 months of well-conducted anticoagulation.^9^, ^10^ Its prevalence is relatively low compared with acute pulmonary embolism, and it is estimated that around 3% of patients surviving the acute event will be diagnosed with CTEPH.^11^ It should be noted that a significant proportion of epidemiological studies were conducted using the old definition of precapillary PH, whereas the current definition is based on 20 mmHg and 2 WU. It is therefore possible that this incidence underestimates the actual incidence based on the new definition. The pathophysiology of CTEPH is complex. History of venous thromboembolism, thrombophilia, and various co-morbidities such as splenectomy, the presence of venous devices, or inflammatory phenomena have been incriminated.^12^, ^13^, ^14^, ^15^, ^16^ Disorders of angiogenesis preventing adequate resolution of blood clots have also been highlighted.^17^, ^18^ The persistence of pulmonary endovascular obstructions will increase PVR and thus RV afterload, which may progress to a maladaptive RV remodeling and RV failure.^19^ The presence of hyperperfused areas, where blood flow is diverted from obstructed to unobstructed zones, will lead to shear stress at the microvascular level, triggering microvasculopathy.^20^ Microvasculopathy is also found in hypoperfused areas and may result from anastomoses with the systemic circulation.^21^ Microvascular damage is characterized at the precapillary level by thickening of the intima and hypertrophy of the media, with muscularization of the arteries.^20^ The capillary and post-capillary sides are not spared either, with capillary hemangiomatosis and venular alterations due to intimal thickening.^21^ Microvasculopathy can contribute to hemodynamic severity and is suspected when there is a discrepancy between visible lesions on imaging and very severe hemodynamics.

## Current treatments in CTEPH

**Figure 1:**
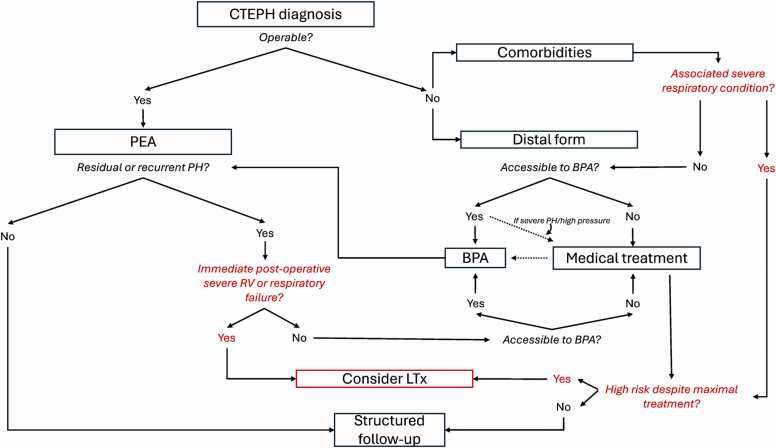
Summarizes the indications of LTx for CTEPH in the modern algorithm treatment of CTEPH. Summary of the potential LTx indications for CTEPH in a simplified modern algorithm treatment for CTEPH.

CTEPH treatment may be divided in 4 components: general treatments, mechanical treatments, medical treatments, and LTx (Figure 1). General treatments include anticoagulation, either vitamin K antagonist (VKA) or direct oral anticoagulant (DOAC), treatment of right heart failure (in particular with diuretics), treatment of respiratory failure with oxygen, and hygienic and dietary measures (rehabilitation, contraception, low-salt diet, etc.).^9^, ^10^ Mechanical treatments include PEA surgery and BPA. These treatments aim to restore pulmonary blood flow through the impaired lung circulation. A full description of these therapeutic modalities has been provided elsewhere.^22^, ^23^ PH medications may also be proposed, although their effects are less well understood. They probably act on microvasculopathy via a vasodilatory effect, a possible anti-remodeling effect, and maybe an effect on cardiac output.^24^ Riociguat, a guanylate cyclase stimulator, has been specifically approved for CTEPH after showing its efficacy in the CHEST study.^1^ Treprostinil and macitentan have also been shown to improve hemodynamics and functional capacity.^2^, ^3^ However, a study evaluating higher doses of macitentan (75 mg, MACiTEPH study, unpublished) was terminated prematurely due to an interim analysis demonstrating futility in continuing the trial.^25^

Recent years have also seen the emergence of combined therapeutic approaches (Figure 1). Most combinations of PEA, BPA, and drug treatments are possible and have been described.^4^, ^26^ The most common cases are bridge to BPA with PH medications, notably based on an ancillary study to the RACE trial demonstrating fewer complications in patients with impaired hemodynamics receiving riociguat before BPA.^27^ Bridging medication can also be proposed in very severe patients (PVR > 10 WU, decreased CI) prior to PEA. Bridging can be performed with monotherapy or combined treatments (dual or triple therapy), although the evidences for combined therapy in CTEPH are scarce.^28^ BPA can also be used to treat residual or recurrent PH after PEA.^29^ Exceptionally, BPA is now even sometimes proposed before PEA, notably to reduce hemodynamic severity, especially if there is one side with predominantly distal lesions and one side with predominantly proximal lesions.^30^ Finally, drug therapy can be used to treat residual or recurrent PH after mechanical treatment.^1^, ^3^

The complexity of this so-called multimodality approach makes it essential to treat these patients in experienced centers with access to the full range of CTEPH treatments. The therapeutic decision is based on multidisciplinary consultation, with different specialties represented and with sufficient experience, such as a surgeon specialized in PEA, interventional radiologists or cardiologists with BPA expertise, dedicated radiologists, and physicians specialized in PH.^4^, ^9^, ^26^

## Treatment failure in CTEPH

Despite all this progress and combined multidisciplinary management, there are still patients for whom an inadequate response or even treatment failure is possible. Based on data from various registries and series, we know that the prevalence of residual PH after PEA is around 25%.^31^ There are less clear-cut data on residual PH after BPA or recurrence after mechanical treatment. However, centers treating CTEPH patients all have a fraction of patients who have not responded to any mechanical treatment and continue to progress and deteriorate clinically and hemodynamically, much like PAH patients. This may particularly concern patients with a mean pulmonary artery pressure greater than 30 mmHg after mechanical treatment.^32^

There are also patients whose distal lesions make them inaccessible to mechanical treatment or whose respiratory comorbidities prevent mechanical treatment. These patients have a significantly poorer prognosis, suggesting that alternative therapeutic options like LTx may have to be considered in selected cases.^5^, ^33^

## Indications of LTx for CTEPH

LTx is the radical surgical treatment of severe pulmonary vascular diseases for well-selected patients. Transplantation for CTEPH is relatively rare. A series of 68 patients transplanted (bilateral LTx or combined heart-LTx) for precapillary PH shows that CTEPH accounts for around 9% of indications in this subgroup, while the vast majority are patients with congenital heart disease or idiopathic PAH.^34^ In another large series, CTEPH represented only 4% of the patients referred to LTx for pre-capillary PH.^35^ It should however be noted that in both studies most patients were transplanted before the advent of BPA and the emergence of drug treatments. Indeed, while in the past, LTx was considered a last resort in patients with CTEPH when other therapies failed; it is now no longer mentioned as an option for CTEPH in the 2022 joint PH guidelines of the European Society of Cardiology (ESC) and the European Respiratory Society (ERS).^36^ Hence, with the advent of BPA and medical therapy in the last decade and the increasing number of centers offering a multimodality approach, CTEPH patients will increasingly be able to avoid LTx. However, despite the fact that absolute numbers of CTEPH patients with an indication for LTx will decrease and its prevalence may become rare, in our opinion, the indication will not completely vanish. LTx for CTEPH may still be considered in the following situations: 1) failure or exclusion of other procedures in patients with persistent PH and the possibility of subsequent deterioration of RV function; 2) inability to wean from extracorporeal membrane oxygenation (ECMO) after PEA; and 3) in some cases of primarily subsegmental disease (University of California, San Diego surgical classification level IV), not amenable for mechanical treatment options.^37^

It should be emphasized that although residual PH after PEA can often be explained by microvasculopathy, not uncommonly, 2 other groups of CTEPH patients also present themselves with residual PH after PEA for which a redo PEA should always be considered and preferred over LTx^1^: patients with incomplete surgical clearance and^2^ patients with true recurrent CTEPH.^38^ The majority of patients within the first group previously underwent their primary PEA in another low-volume CTEPH center.^39^, ^40^, ^41^ This specific finding underscores the importance of expertise on an individual surgeons’ level. The first group will probably become a smaller part of the number of patients that need to be reoperated in the future, since the much less risky BPA can now be used for residual lesions at the segmental and subsegmental level. Through further centralization of care and with growing surgical expertise, it should even disappear completely.^42^ The majority of patients in the second group had at least 1 recognized risk factor for the development of thromboembolic disease with coagulation disorders (especially antiphospholipid syndrome) and poor anticoagulation compliance as major predisposing factors.^39^, ^40^, ^42^ These findings underscore the importance of a meticulous anticoagulation treatment post-PEA.

Reoperative PEA is very rare, and small series have only been reported by high-volume centers in which it constitutes between 0.7% and 1.5% of their experience.^39^, ^40^, ^41^, ^42^ Compared to primary PEA patients, reoperative PEA patients were younger and had rather proximal disease. Reoperative PEA results in a clear improvement of pulmonary hemodynamics; however, not as significant as with primary surgery, and in-hospital mortality rates are higher ranging between 7.7% and 40%.^39^, ^40^, ^41^, ^42^ Given the limited median survival after LTx and the associated immunosuppressive-related complications, the authors plea to always consider redo-PEA in a high-volume center by an experienced PEA surgeon, as this still has the potential to be a truly curative treatment strategy. For patients at the stage of severe right heart failure, the presence of a new pulmonary circulation with a normal RV afterload enables normalization or near-normalization of RV function, thereby evacuating the vital risk. This also explains why LTx alone can be performed and, with rare exceptions, combined heart-LTx is not necessary.

The decision for LTx is essentially based on assessment of the risk without transplantation (estimated survival), compared with the survival rate after transplantation.^43^ Severe PAH patients who have reached the stage of being placed on the transplant list have a much lower survival rate compared to survival after LTx, and therefore if the balance is favorable, with satisfactory post-transplant survival and expected quality of life, and in the absence of contraindications, the indication for LTx can be retained. Different specific criteria have been proposed to refer and list patients with severe PAH (Table 1) and they could be used in CTEPH patients with severe PH associated with RV failure despite optimal treatment by PH-targeted therapy and without mechanical therapeutic options.^44^, ^45^

**Table 1:** Criteria Proposed in 2 Scientific Society (ISHTL, International Society for Heart and Lung Transplantation, PVRi, Pulmonary Vascular Research Institute) to Refer and List Patients for Lung Transplantation for Pulmonary Hypertension

ISHLT	
Criteria to refer PH patient to LTx	Criteria to list PH patient for LTx
• ESC/ERS intermediate or high risk or REVEAL risk score 8 despite appropriate PAH therapy • Significant RV dysfunction despite appropriate PAH therapy • Need for IV or SC prostacyclin therapy • Progressive disease despite appropriate therapy or recent hospitalization for worsening PH • Large and progressive pulmonary artery aneurysms • Signs of secondary liver or kidney dysfunction due to PAH • Potentially life-threatening complications such as recurrent hemoptysis	• ESC/ERS high risk or REVEAL risk score >10 on appropriate PAH therapy, including IV or SC prostacyclin analogues • Progressive hypoxemia • Progressive, but not end-stage, liver or kidney dysfunction due to PAH • Life-threatening hemoptysis

PVRi	
Criteria to refer PH patient to LTx	Criteria to list PH patient for LTx
• ESC/ERS 3 stratum high-risk^a^ • REVEAL 2.0 ≥ 8 (intermediate-risk)^a^ • ESC/ERS 4 stratum intermediate high-risk^a^ • RV dysfunction^a^ • Progression of disease^a^ • PH hospitalization^a^ • Hemoptysis^a^ • WHO functioncal class III-IV^a^ • More than a trivial pericardial effusion^a^ • Cardiac index < 2 L/min/m^2^^a^ • Renal dysfunction^a^ • Hepatic dysfunction^a^ • Require parenteral therapy^b^ • Large/progressive PA aneurysm^b^ • Atrial septostomy^b^ • Coronary compression^b^	• ESC/ERS 3 stratum high-risk^c^ • REVEAL 2.0 ≥ 9 (high-risk)^c^ • ESC/ERS 4 stratum high-risk^c^ • Severe RV dilatation/dysfunction^c^ • Progressive but not end-stage renal disease attributable to PAH^c^ • Progressive but not end-stage hepatic disease attributable to PAH^c^ • Hospitalization for right heart failure^c^ • Life threatening hemoptysis^c^ • Progressive hypoxemia^c^ • On ECMO life support^b^ • On intravenous inotropes^b^ • On vasopressor^b^

Abbreviations: ECMO, extracorporeal membrane oxygenation; ESC, European Society of Cardiology; ERS, European Respiratory Society; LTx, lung transplantation; PAH, pulmonary arterial hypertension; PH, pulmonary hypertension; RV, right ventricle.

a Despite appropriate therapy.

b At any time.

c Despite maximal medical therapy.

Consequently, it is essential to be able to assess survival in CTEPH patients. Given the range of possible mechanical interventions and the multiplicity of combined therapeutic options, there is no mortality risk score developed specifically for the general population of CTEPH patients. The scores developed for PAH have been proposed for CTEPH patients in whom there are no or no longer any mechanical therapeutic options.^46^ They show that the behavior of CTEPH patients with severe PH on medical therapy is relatively similar to that of PAH patients, and outcomes may be adequately predicted using both REVEAL risk or ESC/ERS risk scores,^47^, ^48^ although a complete validation of these scores in CTEPH populations is still awaited. A severe PH with high-risk score on maximum drug therapy, with no mechanical options available, can therefore be considered as an indication for LTx in well-selected CTEPH patients.^37^

Another potential indication for transplantation in patients with CTEPH is concomitant respiratory disease, most often chronic obstructive pulmonary disease or pulmonary fibrosis. Such severe concomitant respiratory pathology may become a contraindication to PEA and/or render BPA ineffective (reperfusion of diseased parenchymal zones). This indication is relatively rare. In these cases, it is essentially the associated respiratory pathology that will concede the indication for LTx. It is also sometimes difficult to assess the degree of vascular obstruction, as scintigraphy and imaging data are more difficult to interpret in this context, and to differentiate between the pulmonary vascular component due to CTEPH itself and that due to the respiratory disease (group 3 PH).

Finally, LTx for CTEPH patients may be urgently considered after failure of mechanical treatment. In the case of PEA, this may be post-operative refractory RV failure with impossibility to wean from ECMO, particularly in cases of advanced microvasculopathy. In the case of BPA, it may be related to post-procedure severe lung injury. In these circumstances, the indication must be weighed carefully. In general, these patients have not been evaluated for LTx and are in a critical situation with a high risk of further organ dysfunction, making post-transplant prognosis potentially poorer. The available data of 4 CTEPH patients transplanted for RV failure after PEA showed a short-term mortality of 75% due to multiple organ failure.^49^ However, although the results described in anecdotal reports of LTx for CTEPH were not always satisfactory, good results may become more frequent since the introduction of the prolonged ECMO strategy for LTx in the setting of PH, which allows time for RV recovery.^50^, ^51^ Therefore, in our opinion, it may still be acceptable to propose LTx in relatively young patients without comorbidities in this acute and desperate setting. Currently in our practice, in patients with high PVR, a targeted therapy is initiated to drop the PVR under 10 WU before any mechanical treatment. If the PVR remains high (>10 WU) a pre-procedural assessment for LTx is performed. In case of failure of a mechanical procedure, selected patients may then be bridged by ECMO to LTx.

In addition to the indications for transplantation, potential risk factors for CTEPH should be considered when assessing post-transplant risk. The presence of an intravascular catheter, a history of splenectomy, inflammatory bowel disease, or a history of osteomyelitis may increase the risk of complications, particularly infectious ones, which must be taken into account. Vascular aberrations, such as collateral circulation that may come from the bronchial arteries but also from the chest wall, the diaphragm, or the coronary circulation, must be taken into account, as well as a secondary well-developed azygos system in the case of occlusion of the inferior caval vein.

## Technical considerations for lung transplantation in CTEPH patients

CTEPH patients are on lifelong therapeutic anticoagulation, either VKA or DOAC.^52^ Anticoagulation should be carefully evaluated for CTEPH patients on the LTx waiting list. To minimize the risk of bleeding, patients should either be transitioned to therapeutic dose of low-molecular weight heparin or reversal agents should be used at the time of LTx.^53^, ^54^, ^55^

In the vast majority of cases, bilateral LTx is preferred over single-LTx and combined heart-lung transplantation (HLTx). Single-LTx is associated with significant limitations in this context, including a higher risk of over-perfusion injury to the grafted lung, leading to increased incidence of primary graft dysfunction (PGD), insufficient RV afterload reduction, and overall inferior clinical outcomes compared to bilateral LTx.^56^ HLTx is not required for most CTEPH patients, as RV dysfunction often reverses following bilateral LTx due to normalization of PVR. Moreover, the availability of heart-lung block donors is limited, resulting in longer waiting times compared to isolated bilateral LTx.^34^

CTEPH patients requiring LTx typically present with severe PH, necessitating the use of mechanical circulatory support.^57^, ^58^ veno-arterial extracorporeal membrane oxygenation (VA-ECMO) is the preferred strategy over cardiopulmonary bypass (CPB), as it leads to lower incidence of PGD, reduced need for blood transfusions, and has overall better outcomes. Use of CPB is necessary in case of HLTx or in case of concomitant heart surgery. Although veno-venous extracorporeal membrane oxygenation has also been described as intraoperative support in selected CTEPH patients,^59^ its use is not recommended for patients with PH according to most recent societal guidelines.^58^ While veno-venous extracorporeal membrane oxygenation can correct hypoxemia and hypercapnia, potentially improving RV function, it does not provide direct RV unloading and will not sufficiently decrease the risk of acute intraoperative RV failure.^60^, ^61^ Prolongation of mechanical circulatory support into the early postoperative period should always be considered in CTEPH patients with severe PH and RV dysfunction to facilitate reverse cardiac remodeling and protect the newly transplanted lungs from hemodynamic stress.^57^, ^62^ An approach using a centrally canulated low resistance oxygenator between the pulmonary artery and the left atrium has also been proposed to bridge patients to LTx after PEA failure.^7^ However, this is a single-center study involving a relatively limited number of patients, and the value of this pulmonary artery and the left atrium strategy in this setting should be further investigated with next-generation artificial lung devices.

Specific anatomical and pathological characteristics of CTEPH patients must be carefully considered during LTx planning. First, patients who have previously undergone PEA may present with some adhesions around the hilum of both lungs. A meticulous hemostasis throughout the procedure is crucial, taking heparin administration into consideration. According to the recent ISHLT guidelines on perioperative ECLS use, a low dose heparin might be considered sufficient for intraoperative VA-ECMO support; however, CTEPH patient population might require VA-ECMO prolongation to allow RV remodeling, leading to potential subsequent heparin re-administration.^58^, ^63^ Second, during PEA, the surgical dissection occurs in the subintimal plane, between the intima and media of the pulmonary artery. Even in the absence of a previous PEA, the dissection around the pulmonary artery might be challenging due to chronic inflammation and loss of natural tissue planes. This can leave the arterial wall structurally weakened, posing risks during hilar dissection and anastomosis in LTx. The same holds true for very rare and theoretical cases in which proximal pulmonary artery disease may still be present. This disease in the pulmonary trunk or just beyond the pulmonary artery bifurcation is ideally removed by the PEA surgeon just before making the anastomosis of LTx and will therefore require CPB and sternotomy. Third, due to chronic obstruction of the pulmonary vasculature, alternative collateral circulation develops in many CTEPH patients, including hypertrophied bronchial arteries.^64^ These vessels can be a source of significant intraoperative bleeding and should be thoroughly evaluated on preoperative computed tomography angiography. Use of CPB instead of VA-ECMO can recuperate this intraoperative blood loss. Embolization of the hypertrophied collateral circulation before the LTx may be considered, although no consensus exists on this point in a recent survey of experts.^45^ Anticipating these challenges is essential for minimizing perioperative risks and optimizing surgical outcomes in this complex patient population. In the absence of proximal CTEPH disease, the clamshell incision remains our preferred approach, as it gives better exposure to the hilus of the lung, avoids a redo-sternotomy with all its associated risks, and gives better exposure to collateral circulation. Hence, meticulous hemostasis is crucial throughout the procedure to control even minor bleeding.

Finally, when LTx is indicated, the procedure should be performed in a high-volume transplant center with specific expertise in the management of CTEPH, given the unique anatomical and physiological considerations associated with this challenging condition.

## Post-operative management

The intraoperative and immediate perioperative periods involve a number of specific risks, including those associated with RV failure, increased risk of PGD, and bleeding.

Higher risk of PGD is driven, to a certain extent, by a sudden increase of left cardiac chamber filling, while the left ventricle suffers from left diastolic dysfunction previously induced by low right-sided CO and compression of the RV in the context of an inelastant pericardial sac. The sudden increase of preload for such a chronic preload-deprived left ventricle can lead to a sudden increase in diastolic left ventricular pressure transduced to the capillary level, resulting in pulmonary edema. Prolonged use of ECMO in the hours or even days following transplantation can reduce this risk of PGD, allowing time to RV to recover and to LV to expanse again.^65^, ^66^

The presence of significant collateral circulation from the systemic circulation and pleural adhesions may also be responsible for significant intra- and postoperative bleeding, necessitating possible revision surgery or embolization. As a result, 75% of the CTEPH patients transplanted with combined HLTx necessitated a revision for bleeding.^34^ Significant bleeding can increase morbidity and the risk of multiple organ failure postoperatively, thereby compromising short-term prognosis.^67^

Particular attention must be paid to anticoagulation in the immediate postoperative period. This should be resumed as soon as possible, given the high risk of recurrence of acute pulmonary embolism in the first few months after LTx.^68^ The risk is due, among other things, to the surgery itself, prolonged immobilization, the presence of multiple venous catheters, the toxicity of the immunosuppressive regimen, and the risk of inflammatory complications such as infections. Early resumption of anticoagulation should always be weighed against the risk of bleeding and discussed with the surgical team. Low-molecular-weight heparins may have an interesting place here, given their ease of use and attractive safety profile. A pragmatic attitude consisting in proposing a prophylactic dose until the risk of bleeding has been eliminated is also legitimate.

## Long-term outcomes and management

Once the immediate critical perioperative phase is over, RV readjustment is rapid, so that after just a few weeks, patients transplanted for CTEPH do not really differ from those transplanted for other indications.

Few series have evaluated outcomes after LTx for CTEPH. A recent Chinese series of 7 patients showed relatively favorable outcomes.^59^ However, patient selection is not clearly described. It appears that some of these patients had associated pulmonary fibrosis. Moreover, it should be noted that the CT scans presented showed that most of the patients were operable but did not undergo surgery, suggesting a lack of accessibility to PEA. One patient probably had undergone emergency LTx on ECMO, and 1 patient benefited from a single LTx, both with relatively favorable short-term outcomes. In a large series of patients referred for LTx for pre-capillary PH, 4 patients were transplanted for CTEPH, with favorable long-term outcomes being a 5-year survival of 100%.^35^ Other data show a 4-year survival of 25% for urgent LTx performed for post-PEA RV failure.^49^ Combined HLTx has also been reported with favorable outcomes.^34^, ^69^

Long-term management is not different from that of other LTx recipients. The risk of recurrence has not really been established, given the rarity of this indication. However, as with all other CTEPH patients, effective anticoagulation should be continued for life. The type and dose of anticoagulation should be carefully selected. VKAs may have an interesting place, given the absence of significant interactions with immunosuppressive drugs and their easy monitoring in patients with regular blood tests. The use of DOACs may also be considered. Particular attention should be paid to the type and dose of DOAC. There is a risk of interaction with immunosuppressive treatments such as cyclosporine or tacrolimus, and the dose needs to be adapted to the renal function, which is often impaired in LTx recipients.^70^ Specialized advice may be required to assess the type, dose, and monitoring of anticoagulation in lung transplant patients.

## Conclusion

Recent years have seen the emergence of new therapeutic modalities for CTEPH, enabling curative treatment in the vast majority of patients. However, there will always be a group of patients who do not respond or for whom the available mechanical treatments cannot be proposed. The criteria developed for transplantation in PAH can be pragmatically transposed to CTEPH patients. In these patients, LTx remains a valid option. There are, however, special surgical precautions for which specific expertise and transplantation in a high-volume center are required.

## Conflicts of Interest statement

The authors have no conflict of interest directly related to the present work.
